# Constructing atomically-dispersed Mn on ZIF-derived nitrogen-doped carbon for boosting oxygen reduction

**DOI:** 10.3389/fchem.2022.969905

**Published:** 2022-08-25

**Authors:** Yaoyao Deng, Jiazheng Pang, Wenzheng Ge, Minxi Zhang, Wentao Zhang, Wei Zhang, Mei Xiang, Quanfa Zhou, Jirong Bai

**Affiliations:** Research Center of Secondary Resources and Environment, School of Chemical Engineering and Materials, Changzhou Institute of Technology, Changzhou, China

**Keywords:** single atom, electrocatalyst, adsorption, active sites, oxygen reduction reaction

## Abstract

Exploring durable and highly-active non-noble-metal nanomaterials to supersede Pt-based nanomaterials is an effective way, which can reduce the cost and boost the catalytic efficiency of oxygen reduction reaction (ORR). Herein, we constructed atomically-dispersed Mn atoms on the ZIF-derived nitrogen-doped carbon frameworks (Mn-N_x_/NC) by stepwise pyrolysis. The Mn-N_x_/NC relative to pure nitrogen-doped carbon (NC) exhibited superior electrocatalytic activity with a higher half-wave potential (*E*_1/2_ = 0.88 V) and a modest Tafel slope (90 mV dec^−1^) toward ORR. The enhanced ORR performance of Mn-N_x_/NC may be attributed to the existence of Mn-N_x_ active sites, which can more easily adsorb intermediates, promoting the efficiency of ORR. This work provides a facile route to synthesize single-atom catalysts for ORR.

## Introduction

The increasingly serious energy crisis has accelerated the growing trend of energy conversion devices. Particularly, proton exchange membrane fuel cells (PEMFCs) exhibit widespread application prospects on account of their high performance and no noxious gas emission, in which oxygen reduction reaction (ORR) is decisive in the overall efficiency of PEMFCs (Wan and Shui, 2022). Currently, platinum (Pt)-based materials are extensively considered as ideal ORR electrocatalysts, (Zhao et al., 2019; Ying, 2021), but their commercial applications are restricted by the shortage and high price of Pt. Therefore, exploration of cost-effective transition-metal electrocatalysts to substitute Pt-based catalysts becomes a critical research direction (Huang et al., 2019; Zhang et al., 2020a; Rao et al., 2022a; Hu et al., 2022; Li et al., 2022; Wang et al., 2022).

Recently, transition-metal single-atom catalysts (SACs) draw wide concern as ORR catalysts on account of their efficient atomic utilization and high tunability of electronic states through tailoring the coordination environment (Wang et al., 2019; Cheng et al., 2020; Shi et al., 2020; Xie et al., 2020; Zhang and Guan, 2020; Zhao et al., 2020; Zhu et al., 2020; Yang et al., 2021a; Zhang et al., 2021a; Yang et al., 2021b; Rao et al., 2022b). Among them, iron (Fe)-based SACs have been popularly studied on account of their excellent electrocatalytic performance in ORR (Li et al., 2019; Ye et al., 2019; Chen et al., 2020; Zhang et al., 2021b; Wang et al., 2021; Rao et al., 2022c; Liu et al., 2022). For example, Zhang et al. successfully constructed Fe-N-C SACs, which exhibited superior catalytic performance in ORR. This is related to the atomically dispersed FeN_4_ active sites and the peculiar 3D porous layered structure (Zhang et al., 2020b). However, the active sites of Fe-SACs have strong adsorption capacity for *OH, resulting in a high energy requirement for *OH desorption from the active sites, reducing the catalytic efficiency (Han et al., 2020). Therefore, the study of transition metal SACs with appropriate adsorbing effectiveness for *OH is an effective method to acquire efficient ORR catalysts. Manganese (Mn) adjacent to Fe on the periodic table has the outer layer electrons of 3d^5^4s^2^. The adsorption energy of Mn-N-C for *OH is lower than that of Fe-N-C, which is consistent with the density functional theory (DFT) analyses (Xiong et al., 2019; Hu et al., 2021; Li et al., 2021; Peng et al., 2021; Zhou et al., 2022). Besides, Mn is among the richest metals on earth. Hence, Mn-based SACs with a Mn-N-C structure are worth studying as potential ORR electrocatalysts on account of abundant reserves and unique electronic structure (Shang et al., 2020a; Han et al., 2021).

Considering that the Mn element is abundant and cheap on earth, and Mn-N-C has comparatively low adsorption energy for *OH, we adopted a stepwise pyrolysis way to construct atomically-dispersed Mn atoms on the ZIF-derived nitrogen-doped carbon (Mn-N_x_/NC) as ORR catalysts. We utilized the aberration-corrected high-angle annular dark-field scanning transmission electron microscopy (AC-HAADF-STEM) to demonstrate that Mn is dispersed as a single atom on the nitrogen-doped carbon (NC). Owing to the sufficient atomically-dispersed Mn-N_x_ active sites, Mn-N_x_/NC exhibits superior electrocatalytic activity and stability toward ORR in alkaline media. Noticeably, Mn-N_x_/NC possesses a higher half-wave potential (*E*_1/2_) over NC, and similar to commercial Pt/C. Moreover, Mn-N_x_/NC exhibits excellent electrochemical durability with almost no loss of activity after 3000 cycles. This work supplies a general method to prepare non-noble-metal SACs for electrochemical applications.

## Experimental section

### Chemicals and materials

Zn(NO_3_)_2_·6H_2_O (AR, 99%), Mn(CH_3_COO)_2_ (MnAc, AR), 2-methylimidazole (2-MeIm, 99%), methanol (GR, 99.7%), isopropanol (AR, 99.7%), and hydrochloric acid (AR, 99.7%) were all produced from Sinopharm Chemical Reagent CO., Ltd.

### Synthesis of nitrogen-doped carbon

NC with a rhombic dodecahedral structure was obtained by pyrolyzing zinc-imidazole frameworks (ZIF-8) at 900°C. Typically, 11.9 g Zn(NO_3_)_2_·6H_2_O and 12.3 g 2-MeIm were dissolved in 150 ml methanol, respectively. Then the two solutions were blended to form ZIF-8 nanocrystals at ambient temperature (Pan et al., 2018). ZIF-8 nanocrystals were successfully prepared after 24 h stirring. The reaction solution was centrifuged, and the precipitate was washed with methanol for several times to remove the impurities. Then the obtained white precipitate was dried in a vacuum oven. The prepared ZIF-8 precursor was pyrolyzed at 900°C in N_2_ condition to form the NC.

### Synthesis of Mn-N_x_/N

Firstly, 300 mg of NC was dispersed in 30 ml isopropanol, then 10 mg of MnAc was added to the above solution. After 2 h of ultrasonic treatment, the suspension was stirred at 40°C until the isopropyl alcohol evaporated entirely. The obtained black power (MnAc/NC) was collected and ground by an agate mortar. The MnAc/NC was annealed at 900°C for 2 h in N_2_ condition. The obtained sample was pickled with 3 M HCl to remove the unstable metal materials, and then repeatedly washed with water until the supernatant became neutral. The black precipitate was dried into black power in a vacuum oven, and then annealed at 900°C for 1 h in N_2_ condition to obtain the final product Mn-N_x_/NC.

## Results and discussion

**SCHEME 1:**
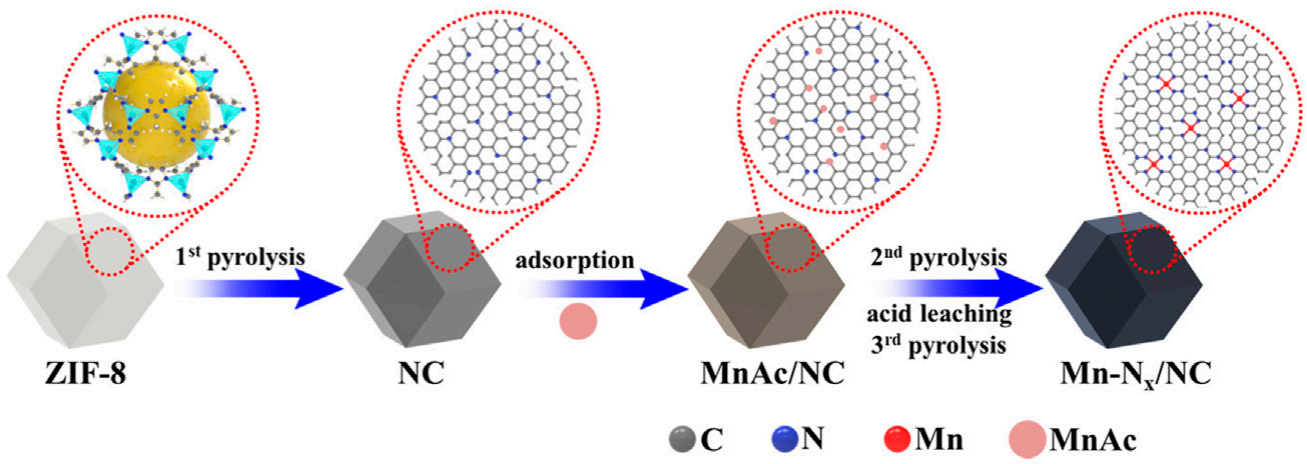
Schematic fabrication process of Mn-N_x_/NC.

Scheme 1 illustrates the route of preparing Mn-N_x_/NC by stepwise pyrolysis. Firstly, ZIF-8 precursors were synthesized (Supplementary Figure S1A), then pyrolyzed at 900°C in N_2_ atmosphere to shape into stable N-doped carbon (NC) structure (Supplementary Figure S1B). NC has the united rhombic dodecahedron shape with the mean size of ∼ 400 nm, which is in agreement with that of ZIF-8. Since the N atoms doped in NC could function as anchors to adsorb MnAc molecules, NC successfully adsorbed MnAc in isopropanol solution to form MnAc/NC (Huo et al., 2020; Qu et al., 2021; Zhai et al., 2022), which was then further pyrolyzed at 900°C. The obtained black power was washed by 3 M HCl solution to etch the unstable metal particles, then annealed again at 900°C for 1 h in N_2_ condition to recover the crystallinity, forming Mn-N_x_/NC which possesses highly-dispersed Mn-N_x_ active sites.

**FIGURE 1:**
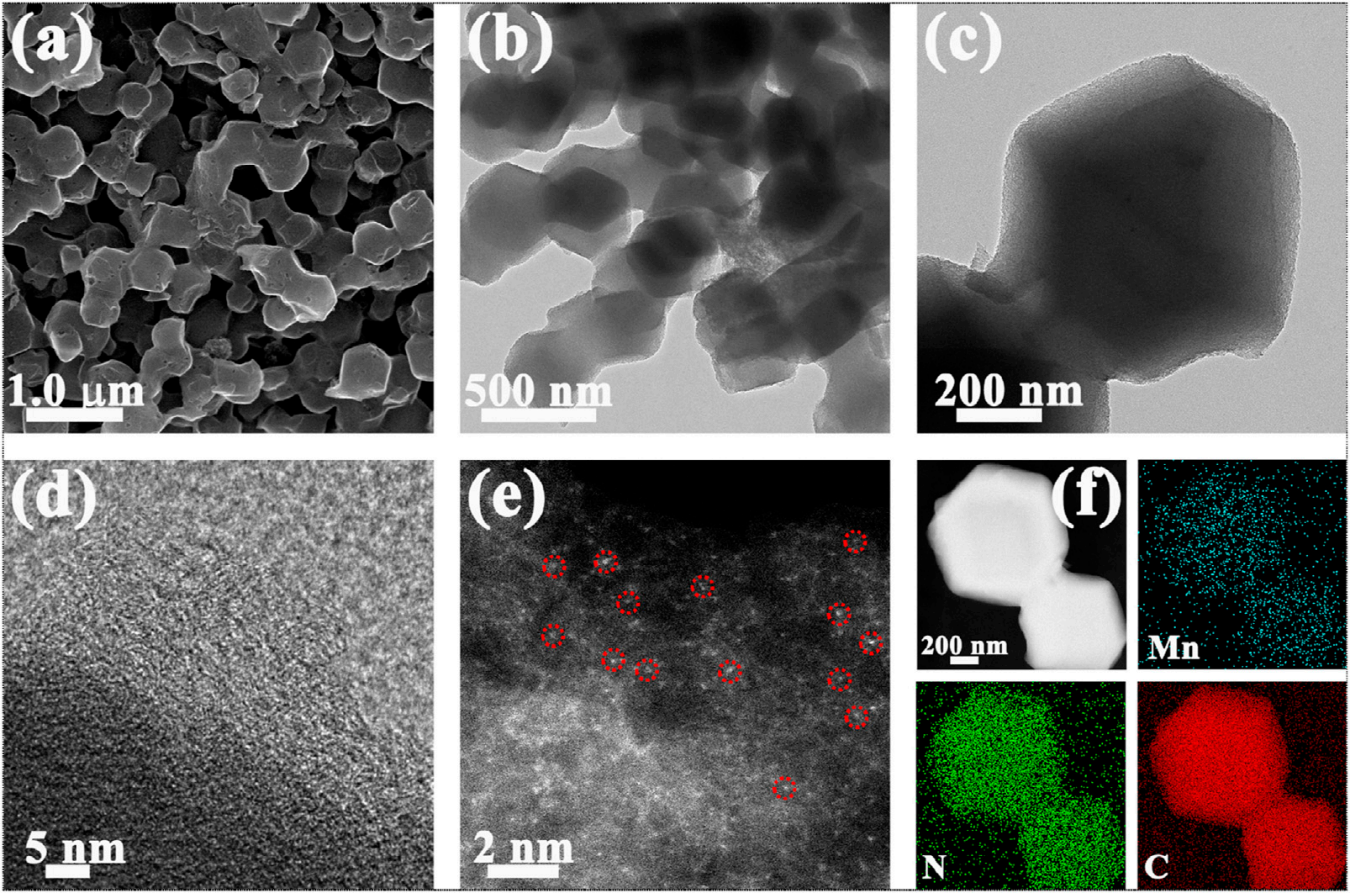
**(A)** SEM, **(B,C)** TEM, **(D)** HRTEM, **(E)** AC-HAADF-STEM images of Mn-N_x_/NC. **(F)** EDX elemental mapping images of Mn-N_x_/NC with Mn (blue), N (green), and C (red).

Scanning electron microscopy (SEM) image exhibits that Mn-N_x_/NC possesses the similar rhombic dodecahedral structure with NC (Figure 1A). Figures 1B,C are transmission electron microscopy (TEM) images of Mn-N_x_/NC with the diameter of ∼ 400 nm, further clearly showing rhombic dodecahedral structure. The high-resolution TEM (HRTEM) image of Mn-N_x_/NC in Figure 1D shows graphitized carbon structure in part. No obvious Mn metal particles in the above results, indicating the presence of highly dispersed Mn-N coordinated structures. Therefore, we utilize the AC-HAADF-STEM technique to explore the existence form of Mn in Mn-N_x_/NC. As shown in Figure 1E, the bright spots are Mn single atoms (partially circled in red), which are atomically dispersed in the NC. Elemental mapping was used to probe into the distributions of Mn, N and C in Mn-N_x_/NC (Figure 1F). The HAADF-STEM image further uncovers that the Mn-N_x_/NC has the similar rhombic dodecahedral structure with NC. The corresponding EDX elemental mapping images exhibit that Mn, N, and C are equally dispersed in the entire structure, which facilitate the possible coordination of Mn and N components. Besides, we further quantified the C, N and Mn content of Mn-N_x_/NC that the Mn mass loading is about 0.81% (Supplementary Table S1).

**FIGURE 2:**
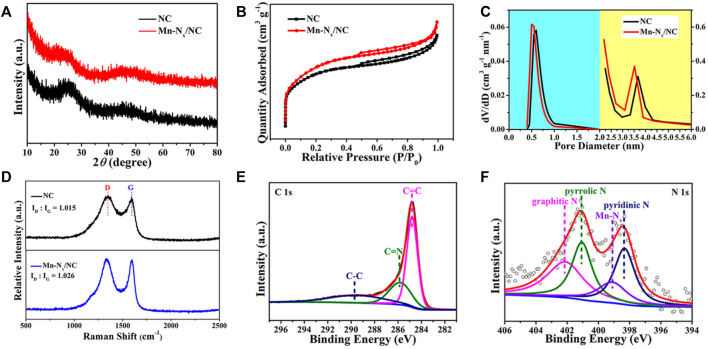
**(A)** XRD patterns, **(B)** N_2_ adsorption/desorption isotherms, and **(C)** homologous pore distribution of NC (in black) and Mn-N_x_/NC (in red). **(D)** Raman spectra of NC and Mn-N_x_/NC. XPS survey spectra for **(E)** C 1s, and **(F)** N 1s of Mn-N_x_/NC.

Structure and phase state of the catalysts were investigated by XRD. The XRD patterns of NC (in black) and Mn-N_x_/NC (in red) showed only two wide shoulder peaks at ∼26^o^ and ∼44^o^ (Figure 2A), which are consistent with the (002) and (010) planes of graphitic carbon (Lin et al., 2019). Besides, the XRD pattern of Mn-N_x_/NC showed no typical peaks of Mn or its compounds, indicating the inexistence of any Mn-based particles. To study the specific surface areas and porosity structures of samples, the N_2_ adsorption/desorption experiments were utilized to test NC and Mn-N_x_/NC. As shown in Figure 2B, the BET specific surface areas of NC and Mn-N_x_/NC are 1064.531 and 1014.221 m^2^ g^−1^, respectively. Compared with NC, the BET specific surface area of Mn-N_x_/NC decreases owing to the adsorption of MnAc (Liu et al., 2021). The NC and Mn-N_x_/NC are mainly micropores (about 0.5 nm), in which Mn-N_x_/NC still keeps the abundant micropores after adsorption of MnAc (Figure 2C). Raman spectroscopy can effectively characterize the disorderly and orderly crystal structures of carbon materials. It can be seen from the Figure 2D that there are two bands at 1350 and 1590 cm^−1^, which matched with disordered carbon (D band) and graphitic carbon (G band), respectively (He et al., 2020; Gharibi et al., 2022; Zhang et al., 2022). It can be concluded that the relative intensity ratios of the D and G band (*I*_D_/*I*_G_) of NC and Mn-N_x_/NC are 1.015 and 1.026, respectively, suggesting the formation of more defects in N-doped carbon after introduction of Mn, which helps enhance the electrocatalytic activity (Rao et al., 2022d; Zou et al., 2022).

Furthermore, we utilize X-ray photoelectron spectroscopy (XPS) to study the elemental composition and valence state of the Mn-N_x_/NC. Figure 2E is the high-resolution C 1s spectrum of Mn-N_x_/NC, which was fitted into three peaks at 284.7 eV (C=C), 285.8 eV (C=N) and 289.4 eV (C-C), respectively (Shang et al., 2020b; Gu et al., 2022). Figure 2F shows the N 1s spectrum of Mn-N_x_/NC, which comprises four main peaks of graphitic N (402.1 eV), pyrrolic N (401.1 eV), pyridinic N (398.3 eV), and Mn-N_x_ (399.1 eV), respectively (Han et al., 2020). The above results manifest the existence of pyridinic N, which possessed lone pair electrons and exhibits stronger activity than graphitic N and pyrrolic N in NC (Qu et al., 2021; Meng et al., 2022). Besides, the Mn 2p spectrum of Mn-N_x_/NC was shown in Supplementary Figure S2, which contains two peaks of Mn 2p_3/2_ and Mn 2p_1/2_. These results demonstrate that the Mn-N_x_/NC has been successfully prepared and is a promising ORR electrocatalyst owing to the sufficient Mn-N_x_ active sites.

**FIGURE 3:**
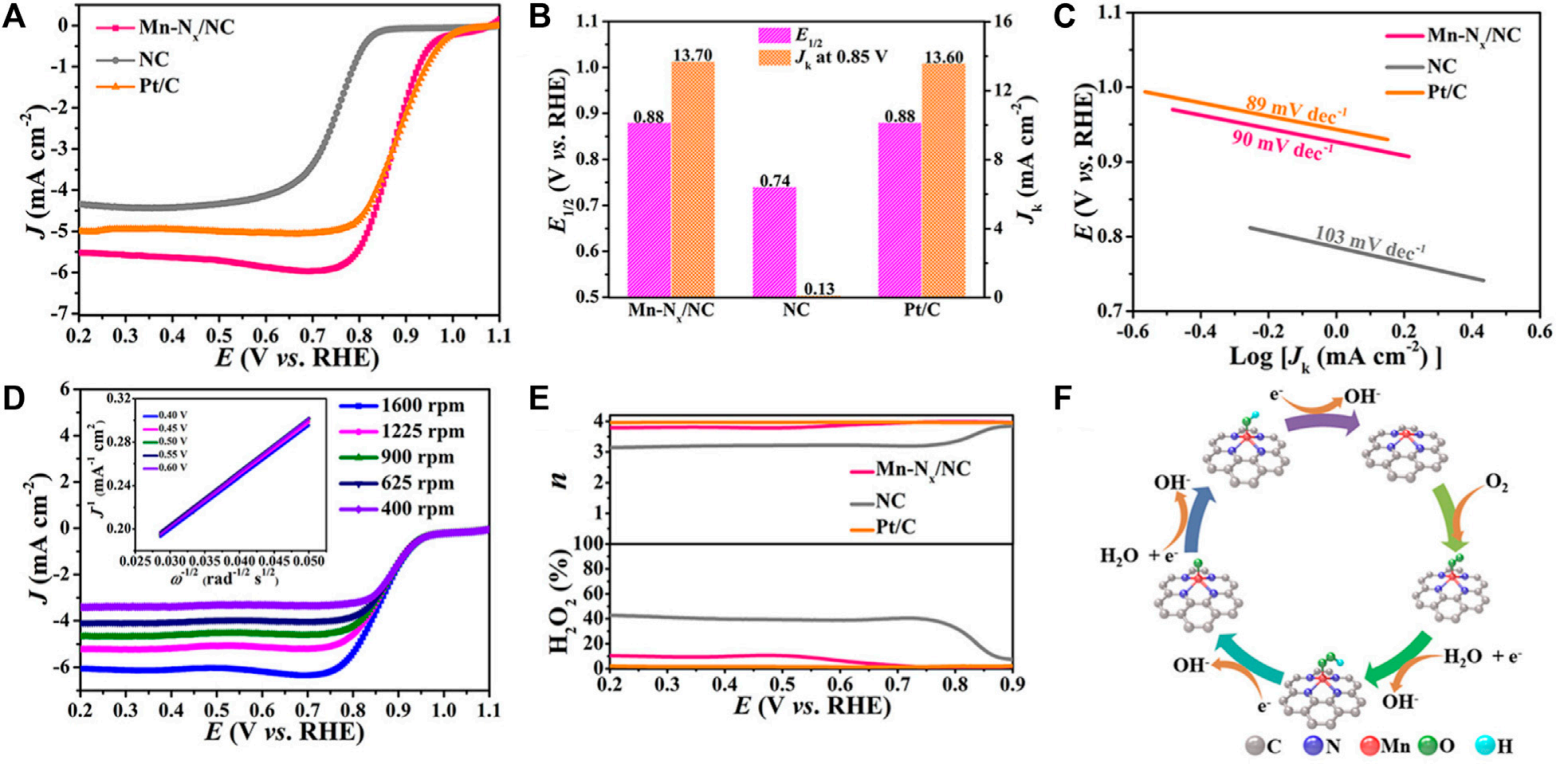
Electrocatalytic performance in ORR. **(A)** LSV curves, **(B)** *E*_1/2_ and *J*_k_, and **(C)** Tafel slopes of Mn-N_x_/NC, NC and Pt/C. **(D)** The LSV curves of Mn-N_x_/NC at varying rotating speeds, K-L plots in the inset. **(E)** *n* and H_2_O_2_ yield of Mn-N_x_/NC and the references. **(F)** Proposed ORR processes on Mn-N_x_/NC.

To prove that Mn-N_x_/NC has excellent electrocatalytic performance, the electrochemical properties of Mn-N_x_/NC were tested by a typical three-electrode system. The liner sweep voltammetry (LSV) curves of Mn-N_x_/NC, NC and commercial Pt/C at a rotating speed of 1600 rpm are shown in Figure 3A, indicating that NC has poor catalytic activity before adsorption of Mn. After the adsorption of Mn, the *E*_1/2_ of Mn-N_x_/NC (0.88 V) is obviously better than that of NC (0.74 V), and was consistent with the extensively used commercial Pt/C (0.88 V) (Figure 3B). Moreover, the kinetic current density (*J*_k_) of Mn-N_x_/NC at 0.85 V is 105.4 times that of NC (13.7 vs. 0.13 mA cm^−2^), suggesting that Mn-N sites may take significant roles in the ORR than the N-C sites (Figure 3B). Mn-N_x_/NC catalyst has lower cost and higher catalytic activity, implying that Mn-N_x_/NC has a good application prospect in ORR electrocatalysis.

The ORR kinetics of the catalysts were explored by the Tafel plots. As shown in Figure 3C, the Tafel slope of Mn-N_x_/NC is 90 mV dec^−1^, which is lower than that of NC (103 mV dec^−1^) and verge on that of commercial Pt/C (89 mV dec^−1^), indicating the faster electron transfer rate of Mn-N_x_/NC in ORR. To further probe into the ORR kinetics of Mn-N_x_/NC, its LSV curves were tested at different rotating speeds. The limiting current density of Mn-N_x_/NC rises proportionally with the increment of rotating speed (Figure 3D). The Koutecky-Levich (K-L) plots of Mn-N_x_/NC reveal superior linearity at potentials of 0.40, 0.45, 0.50, 0.55 and 0.60 V (inset in Figure 3D). The computed electron-transfer number (*n*) of Mn-N_x_/NC between 0.4 and 0.6 V is about 3.86, which is verge on the *n* of commercial Pt/C. (Chen et al., 2022; Xili et al., 2022). Moreover, the electron-transfer mechanism of the catalysts was further researched by utilizing rotating ring disk electrode (RRDE) measurement. Notably, Mn-N_x_/NC has an ignorable ring current (*I*_r_) relative to its disk current (*I*_d_), suggesting its H_2_O_2_ production was basically inhibited during the ORR (Supplementary Figure S3). According to the values of *I*_d_ and *I*_r_, the *n* of Mn-N_x_/NC was computed to be 3.80–3.98 at the potentials from 0.2 to 0.9 V, and the H_2_O_2_ yield was less than 10%, which are analogous to the data of commercial Pt/C (Figure 3E). The above results confirm that Mn-N_x_/NC has a four-electron transfer pathway in ORR process. The proceeding four-electron ORR pathway is shown in Figure 3F, exhibiting that Mn-N_x_ site creats a favorable chemical environment for adsorption of reaction intermediates.

**FIGURE 4:**
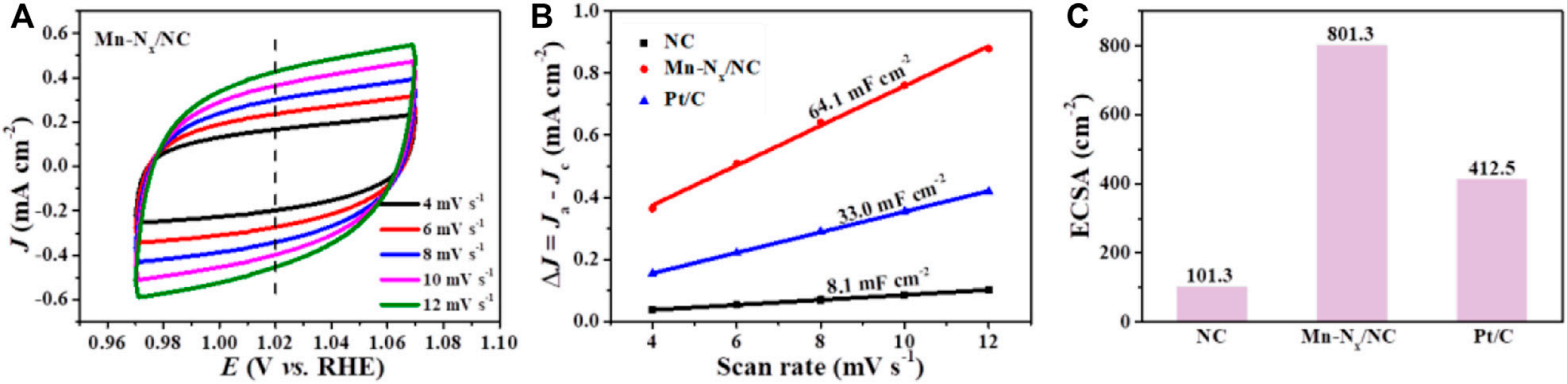
**(A)** CV curves of Mn-N_x_/NC in N_2_-saturated 0.1 M KOH solution at different scan rates from 4 to 12 mV s^−1^. **(B)** Plots density against scan rates for NC, Mn-N_x_/NC and commercial Pt/C. **(C)** The comparison of electrochemical active surface area (ECSA) of NC, Mn-N_x_/NC and commercial Pt/C.

To explore why Mn-N_x_/NC exhibits better activity for ORR, the electrochemical double layer capacitance (*C*_dl_) of the catalysts were investigated, which is a reasonable indicator of electrochemical active surface areas (ECSAs). The *C*_dl_ was computed by plotting cyclic voltammetry (CV) curves in a non-faradaic zone at scan rates from 4 to 12 mV s^−1^ (Figure 4A; Supplementary Figure S4). The Mn-N_x_/NC has a larger *C*_dl_ (64.1 mF cm^−2^) than NC (8.1 mF cm^−2^) as exhibited in Figure 4B. Correspondingly, Mn-N_x_/NC possesses a greater ECSA than NC (Figure 4C), indicating that Mn-N_x_/NC exposes more catalytic sites at the solid-liquid interface, and benefiting the diffusing of oxygen and electrolyte onto Mn-N_x_ active species.

**FIGURE 5:**
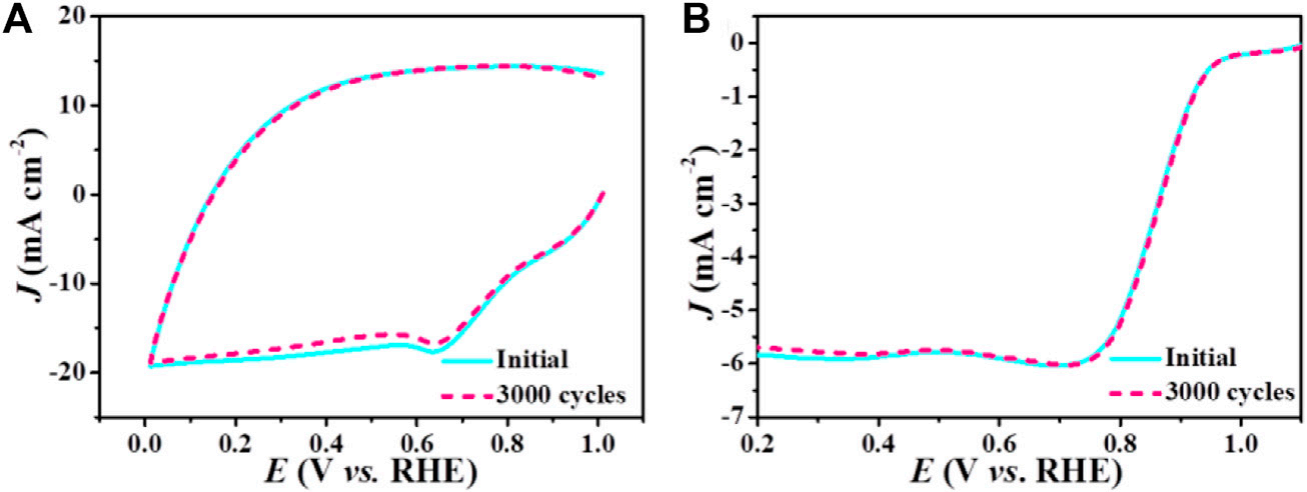
**(A)** CV curves and **(B)** LSV curves of Mn-N_x_/NC before and after 3000 cycles in O_2_-saturated 0.1 M KOH solution.

Except for the electrocatalytic activity, stability is another important criterion to assess ORR electrocatalysts. The stability of Mn-N_x_/NC and commercial Pt/C were evaluated by CV cycling at a scan rate of 200 mV s^−1^ for 3000 cycles (Figure 5A; Supplementary Figure S5A). Compared with commercial Pt/C (Supplementary Figure S5B), the *E*_1/2_ and limiting current density of Mn-N_x_/NC (Figure 5B) changed very little from the initial LSV curves after the 3000 cycles, demonstrating its superior stability during the ORR. Therefore, all of the above analyses confirm that Mn-N_x_/NC exhibit superior electrocatalytic properties toward ORR.

## Conclusion

Generally speaking, we successfully constructed atomically-dispersed Mn atoms on the ZIF-derived nitrogen-doped carbon by a stepwise pyrolysis strategy. The Mn-N_x_/NC exhibited superior ORR performance, which might be related to the formation of Mn-N_x_ active sites and ZIF-derived NC. Mn-N_x_ active sites more easily adsorb intermediates and promote ORR efficiency. ZIF-derived NC with porous structure can supply adequate accessible active sites. Besides, the NC from high temperature pyrolysis has strong corrosion resistance and stability. The obtained Mn-N_x_/NC catalyst possess superior catalytic performance that exhibit higher half-wave potential (*E*_1/2_ = 0.88 V vs. RHE) and excellent stability for the ORR in alkaline media. This work presents new insights for rationally designing structurally-optimized and highly-dispersed catalysts, thus improving the catalytic performance for sustainable energy conversion and generation.

## Data Availability

The original contributions presented in the study are included in the article/Supplementary Material, further inquiries can be directed to the corresponding author.
