# Supplementary material for: Constructing atomically-dispersed Mn on ZIF-derived nitrogen-doped carbon for boosting oxygen reduction
Source: Front Chem. 2022 Aug 25;10:969905. doi: 10.3389/fchem.2022.969905 (PMC9454009; doi:10.3389/fchem.2022.969905)
Supplement: Supplementary file 1 [file DataSheet1.doc]

Supporting Information

Constructing Atomically-Dispersed Mn on ZIF-Derived Nitrogen-Doped Carbon for Boosting Oxygen Reduction

Yaoyao Deng^a^, Jiazheng Pang^a^, Wenzheng Ge^a^, Minxi Zhang^a^, Wentao Zhang^a^, Wei Zhang^a^, Mei Xiang^a^, Quanfa Zhou^a^, Jirong Bai*^,a^

^a^ Research Center of Secondary Resources and Environment, School of Chemical Engineering and Materials, Changzhou Institute of Technology, Changzhou 213032, China. E-mail:baijr@cit.edu.cn

**Materials characterization**

The morphologies of the samples as-synthesized were examined by a scanning electron microscopy (SEM, Hitachi S-4700), a transmission electron microscopy (TEM, TecnaiG220, FEI), and an aberration-corrected high-angle annular dark-field scanning transmission electron microscopy (AC-HAADF-STEM, JEM-ARM300F, 300 kV, Japan). X-ray diffraction (XRD) was performed on a X’Pert-Pro MPD diffractometer (Netherlands PANalytical) with a Cu Kα X-ray source (λ = 1.540598 Å). X-ray photoelectron spectroscopy (XPS, Escalab250Xi, UK) was conducted with a hemispherical electron energy analyzer. Raman tests were performed on a Bruker RAM Ⅱ microscoped system at 532 nm. The N_2_ adsorption/desorption isotherms of Mn-N_x_/NC were investigated using a gas adsorption analyzer (Jingwei Gaobo JWBK200B, China).

**Electrochemical measurement**

Electrochemical measurements were performed by a Pine electrochemical workstation (Pine Research Instrument, USA) using a three-electrode cell. A graphite rod and a saturated calomel electrode (SCE) served as the counter electrode and the reference electrode, respectively. A polished glassy carbon rotating disk electrode (RDE, with an area of 0.196 cm^2^) or a rotating ring disk electrode (RRDE, with an area of 0.245 cm^2^) was applied as the working electrode. The catalyst ink was prepared by ultrasonically mixing 5 mg of a catalyst with 485 µL of isopropanol and 15 µL of Nafion (0.5 wt.%) for 1 h. After that, 10 µL of the ink was deposited on the GC electrode and dried at room temperature. Potentials were referenced to a reversible hydrogen electrode (RHE): *E*_RHE_ = *E*_SCE_ + 0.241 + 0.059 × pH. The liner sweep voltammetry (LSV), cyclic voltammetry (CV) were conducted in N_2_ and O_2_-saturated 0.1 M KOH at room temperature.

The Koutecky-Levich (K-L) equation is given as follows to compute the electron transfer number (n):

1/*J* = 1/*J*_L_ + 1/*J*_K_ = 1/(*Bω*^1/2^) + 1/*J*_K_ (1)

*B* = 0.2*nF*(*C*_O2_) (*D*_O2_)^2/3^υ^-1/6^ (2)

where *J*, *J*_K_, and *J*_L_ are the tested, kinetic, and limiting diffusion current densities, respectively. *ω* is the rotating speed of RDE and *F* is the Faraday constant (96,485 C∙mol^-1^). *C*_O2_, *D*_O2_ and υ correspond to the bulk concentration of oxygen (1.2 × 10^-6^ mol∙cm^-3^), the diffusion coefficient of oxygen (1.9 × 10^-5^ cm^2^∙s^-1^) and the kinematic viscosity of the electrolyte (0.01 cm^2^∙s^-1^), respectively.


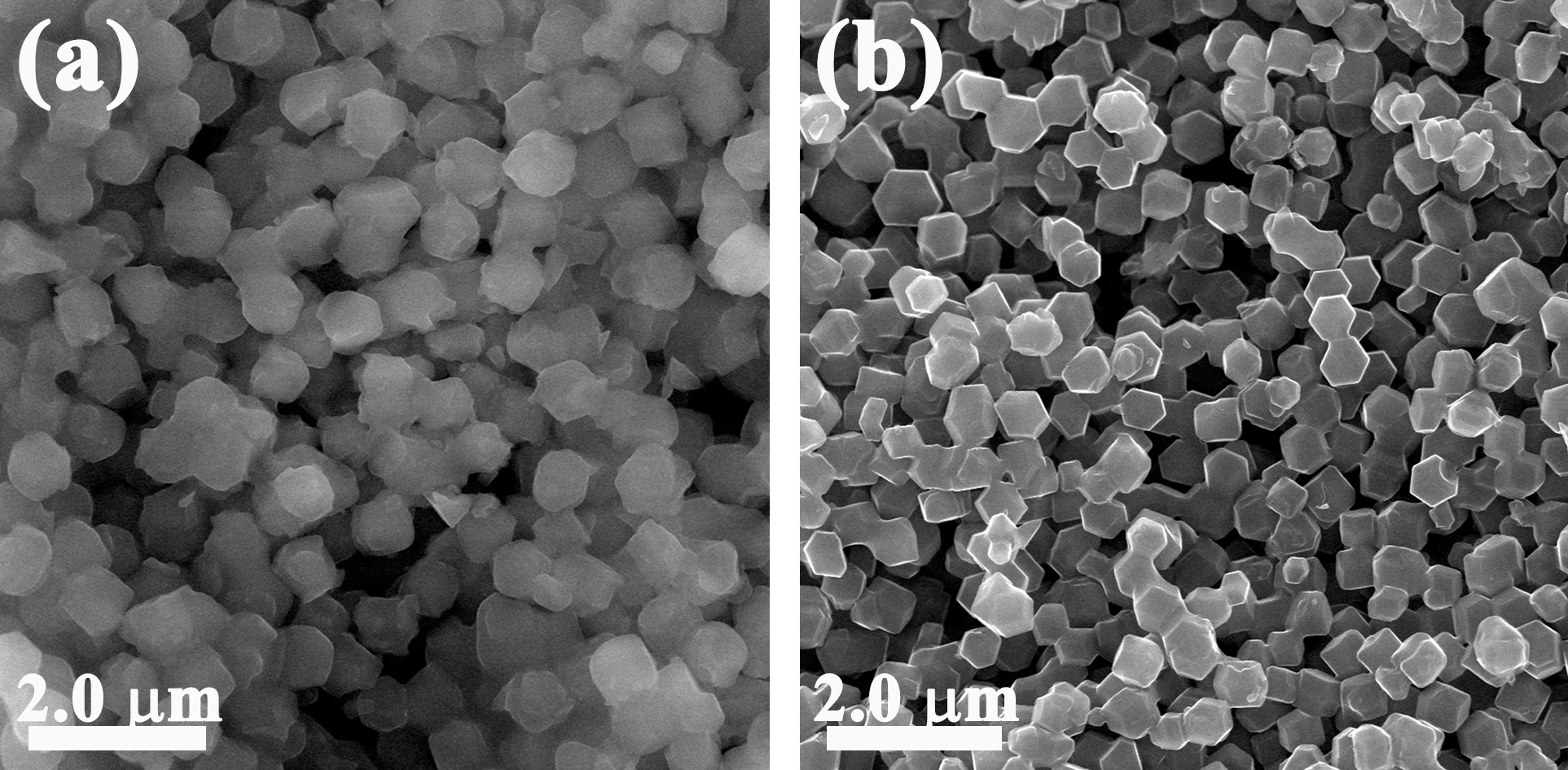


Figure S1 SEM images of (a) ZIF-8 precursor and (b) NC.


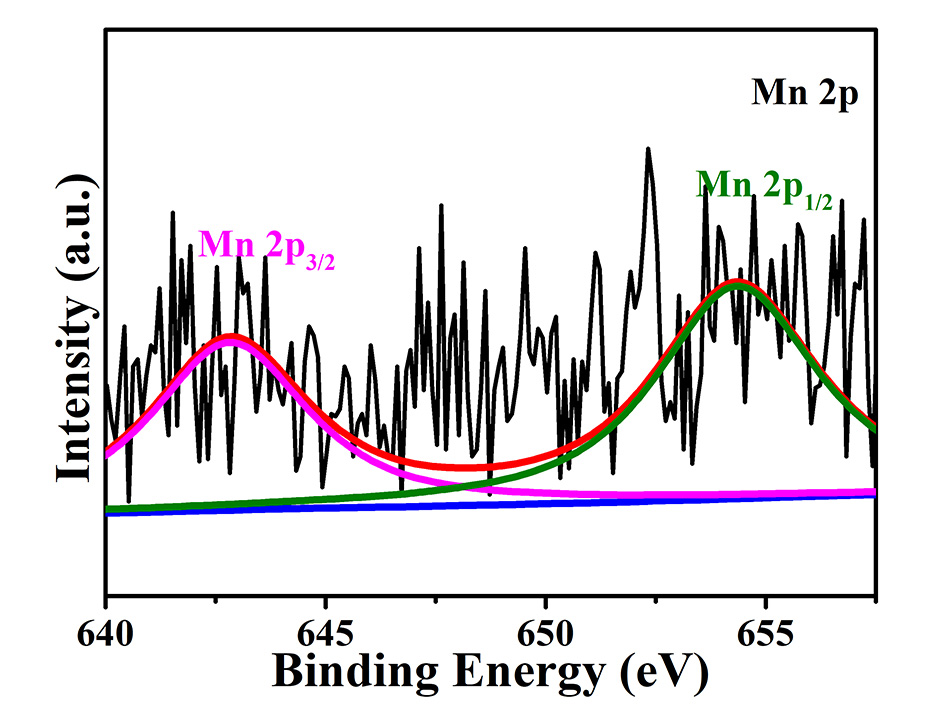


Figure S2 XPS survey spectrum for Mn 2p of Mn-N_x_/NC.


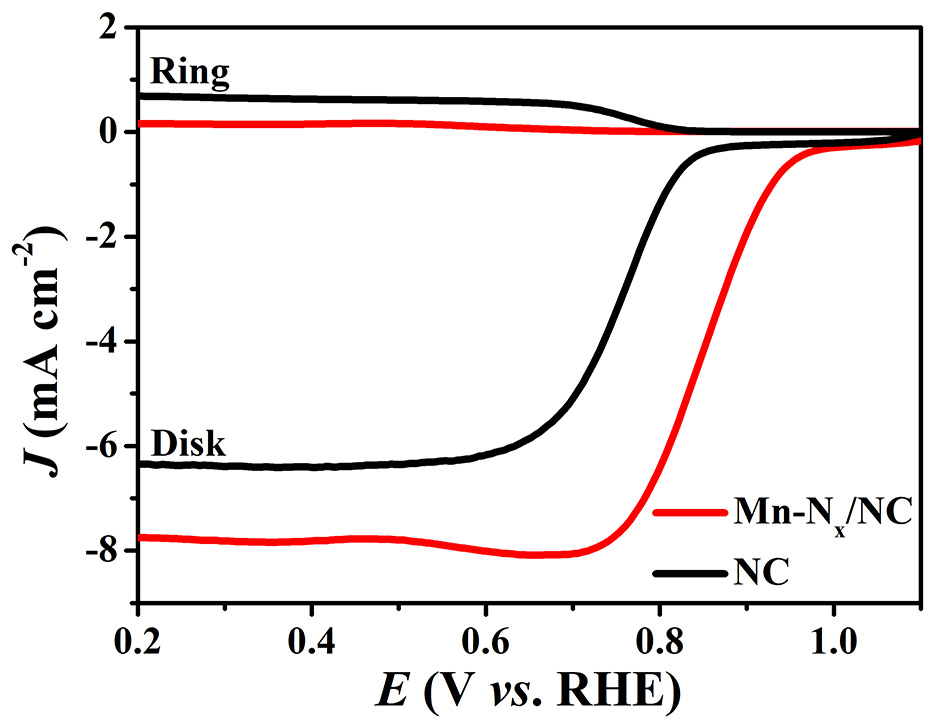


Figure S3 RRDE tests of Mn-N_x_/NC and NC.


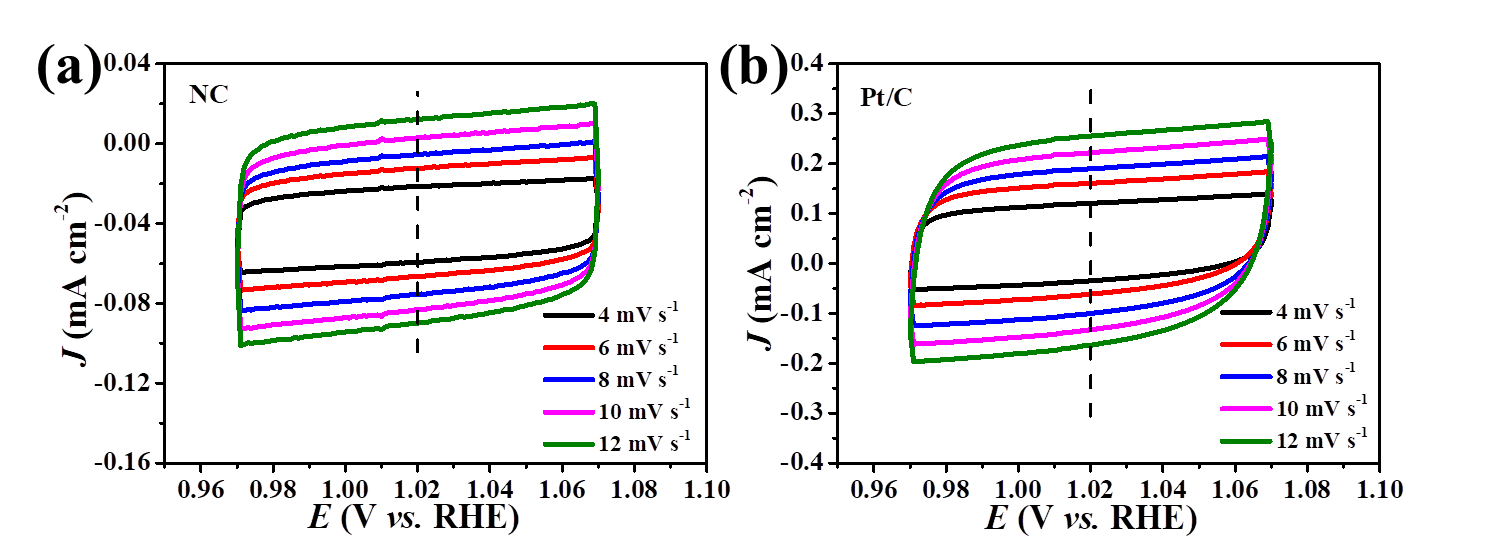


Figure S4 (a) CV curves of NC in N_2_-saturated 0.1 M KOH solution at different scan rates from 4 to 12 mV s^-1^. (b) CV curves of Pt/C in N_2_-saturated 0.1 M KOH solution at different scan rates from 4 to 12 mV s^-1^.


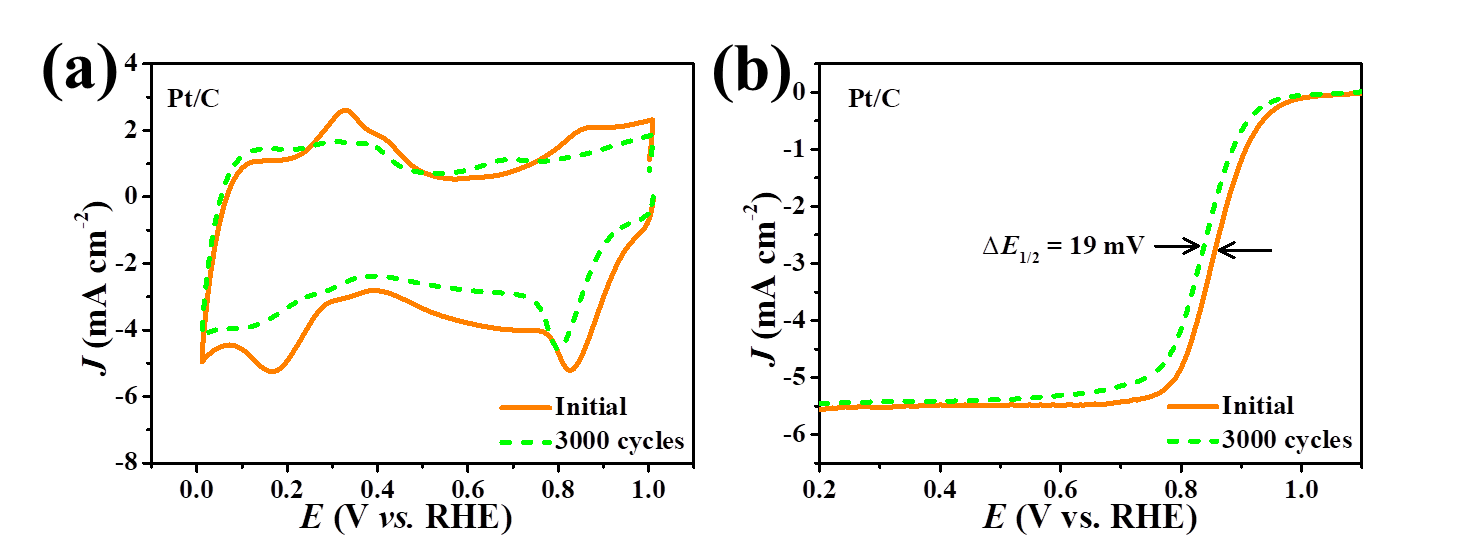


Figure S5 (a) CV curves and (b) LSV curves of commercial Pt/C before and after 3000 cycles in O_2_-saturated 0.1 M KOH solution.

**Table S1.** The compositions of the Mn-N_x_/NC obtained by EDX analysis.

|  | **C (mass%)** | **N (mass%)** | **Mn (mass%)** |
| --- | --- | --- | --- |
| Mn-N_x_/NC | 98.40 | 0.79 | 0.81 |
